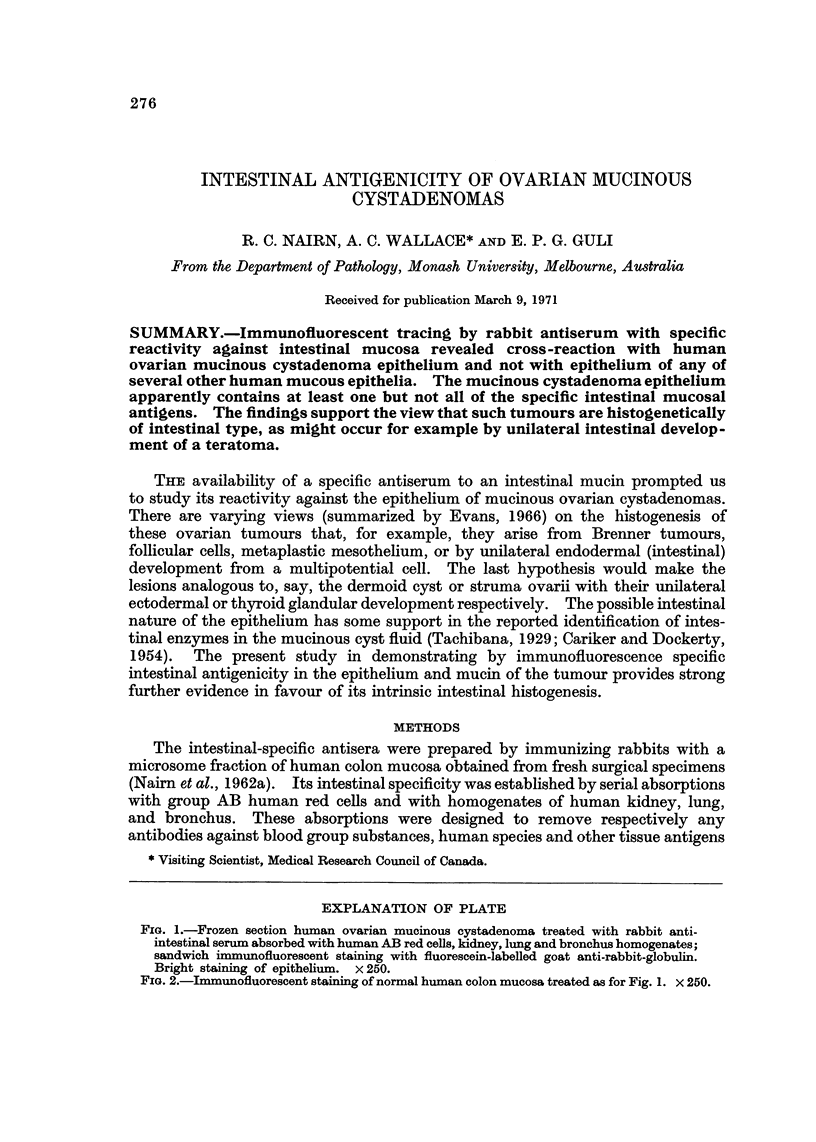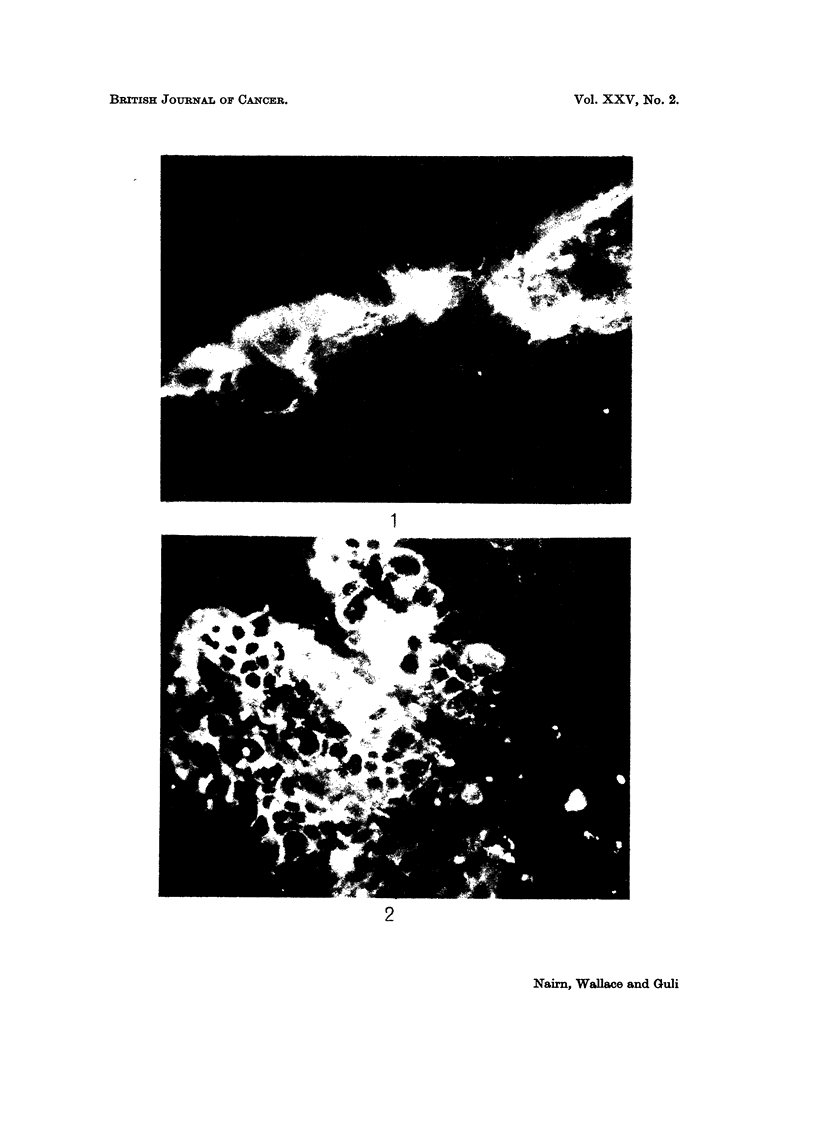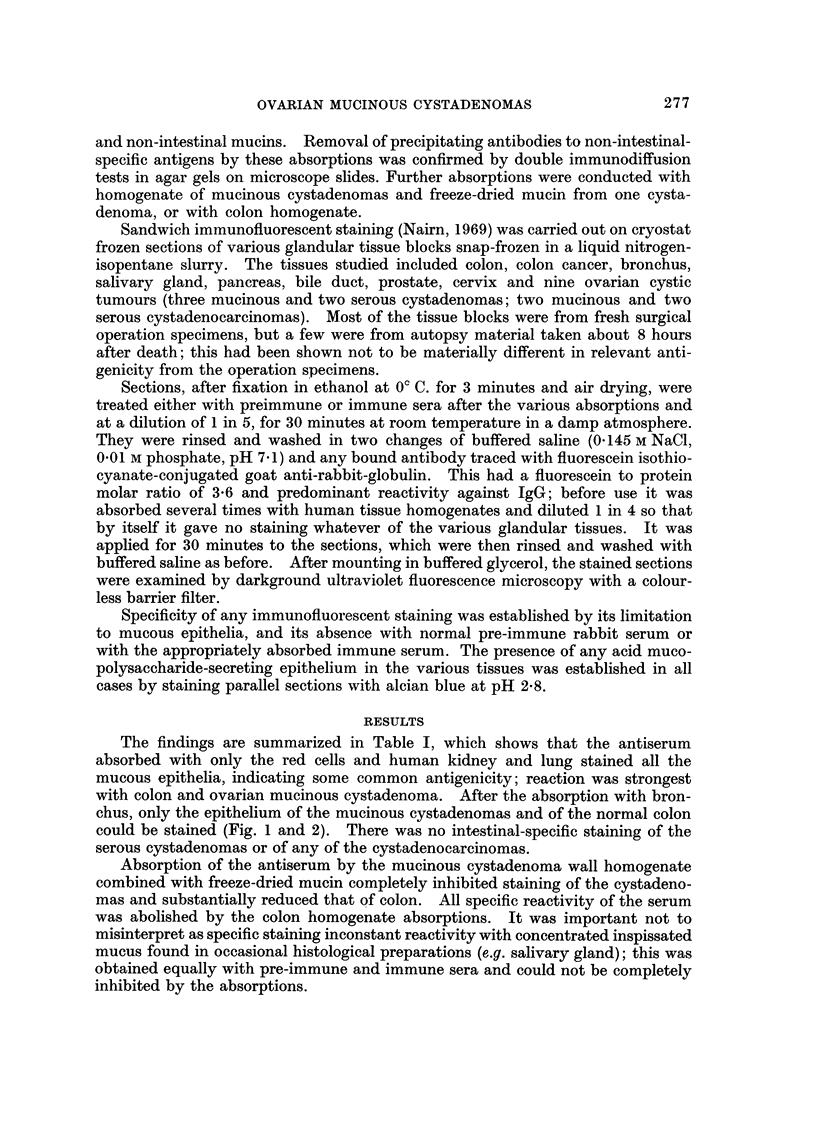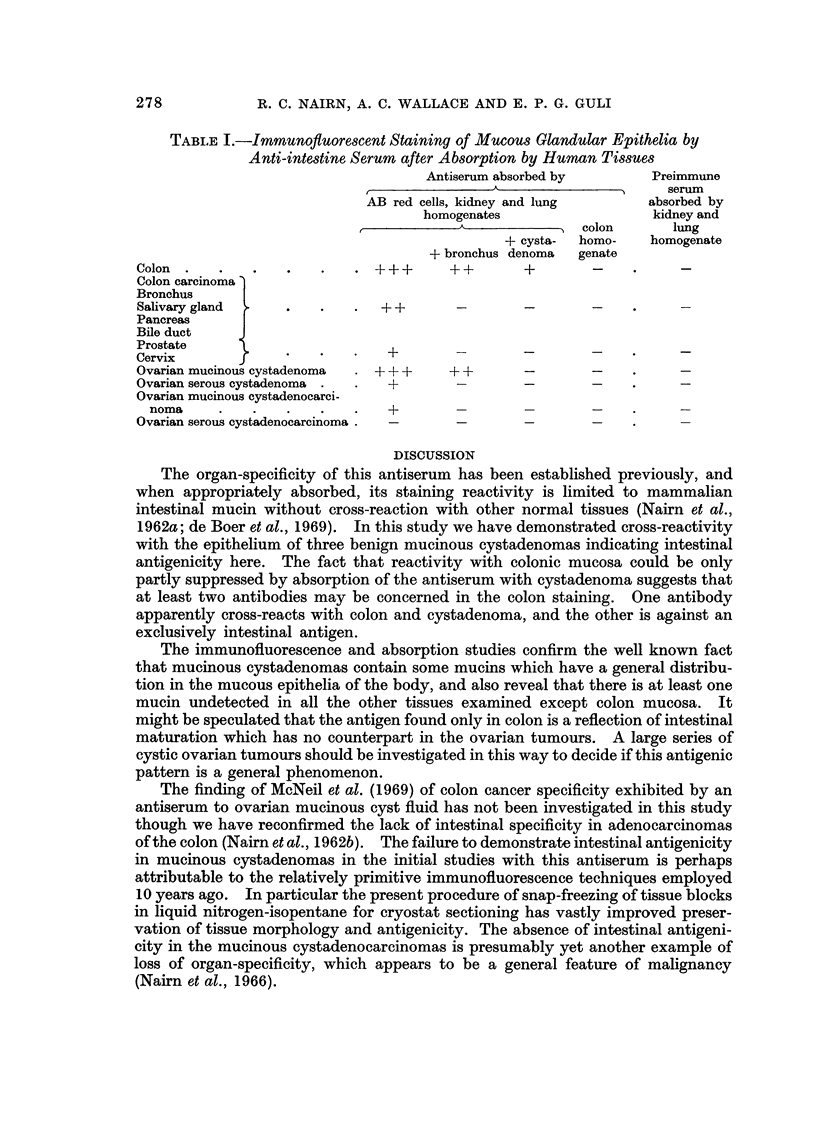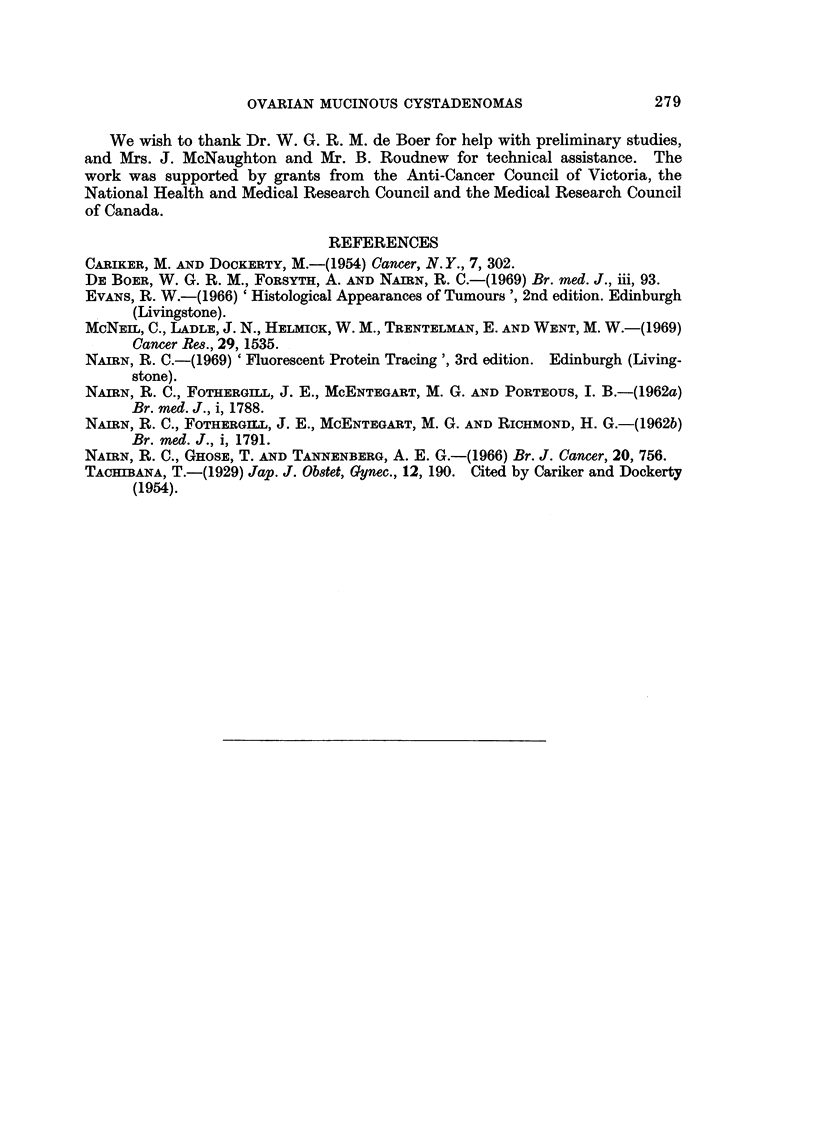# Intestinal Antigenicity of Ovarian Mucinous Cystadenomas

**DOI:** 10.1038/bjc.1971.34

**Published:** 1971-06

**Authors:** R. C. Nairn, A. C. Willace, E. P. G. Guli

## Abstract

**Images:**


### INTESTINAL ANTIGENICITY OF OVARIAN MUCINOUS CYSTADENOMAS

R. C. NAIRN, A. C. WALLACE* AND E. P. G. GULI

*From the Department of Pathology, Monash University, Melbourne, Australia*

Received for publication March 9, 1971

**SUMMARY.—Immunofluorescent tracing by rabbit antiserum with specific reactivity against intestinal mucosa revealed cross-reaction with human ovarian mucinous cystadenoma epithelium and not with epithelium of any of several other human mucous epithelia. The mucinous cystadenoma epithelium apparently contains at least one but not all of the specific intestinal mucosal antigens. The findings support the view that such tumours are histogenetically of intestinal type, as might occur for example by unilateral intestinal development of a teratoma.**

THE availability of a specific antiserum to an intestinal mucin prompted us to study its reactivity against the epithelium of mucinous ovarian cystadenomas. There are varying views (summarized by Evans, 1966) on the histogenesis of these ovarian tumours that, for example, they arise from Brenner tumours, follicular cells, metaplastic mesothelium, or by unilateral endodermal (intestinal) development from a multipotential cell. The last hypothesis would make the lesions analogous to, say, the dermoid cyst or struma ovarii with their unilateral ectodermal or thyroid glandular development respectively. The possible intestinal nature of the epithelium has some support in the reported identification of intestinal enzymes in the mucinous cyst fluid (Tachibana, 1929; Cariker and Dockerty, 1954). The present study in demonstrating by immunofluorescence specific intestinal antigenicity in the epithelium and mucin of the tumour provides strong further evidence in favour of its intrinsic intestinal histogenesis.

### METHODS

The intestinal-specific antisera were prepared by immunizing rabbits with a microsome fraction of human colon mucosa obtained from fresh surgical specimens (Nairn *et al.*, 1962a). Its intestinal specificity was established by serial absorptions with group AB human red cells and with homogenates of human kidney, lung, and bronchus. These absorptions were designed to remove respectively any antibodies against blood group substances, human species and other tissue antigens

* Visiting Scientist, Medical Research Council of Canada.

---

### EXPLANATION OF PLATE

FIG. 1.—Frozen section human ovarian mucinous cystadenoma treated with rabbit anti-intestinal serum absorbed with human AB red cells, kidney, lung and bronchus homogenates; sandwich immunofluorescent staining with fluorescein-labelled goat anti-rabbit-globulin. Bright staining of epithelium. ×250.

FIG. 2.—Immunofluorescent staining of normal human colon mucosa treated as for Fig. 1. ×250.

Nairn, Wallace and Guli and non-intestinal mucins. Removal of precipitating antibodies to non-intestinal-specific antigens by these absorptions was confirmed by double immunodiffusion tests in agar gels on microscope slides. Further absorptions were conducted with homogenate of mucinous cystadenomas and freeze-dried mucin from one cystadenoma, or with colon homogenate.

Sandwich immunofluorescent staining (Nairn, 1969) was carried out on cryostat frozen sections of various glandular tissue blocks snap-frozen in a liquid nitrogen-isopentane slurry. The tissues studied included colon, colon cancer, bronchus, salivary gland, pancreas, bile duct, prostate, cervix and nine ovarian cystic tumours (three mucinous and two serous cystadenomas; two mucinous and two serous cystadenocarcinomas). Most of the tissue blocks were from fresh surgical operation specimens, but a few were from autopsy material taken about 8 hours after death; this had been shown not to be materially different in relevant antigenicity from the operation specimens.

Sections, after fixation in ethanol at 0° C. for 3 minutes and air drying, were treated either with preimmune or immune sera after the various absorptions and at a dilution of 1 in 5, for 30 minutes at room temperature in a damp atmosphere. They were rinsed and washed in two changes of buffered saline (0·145 M NaCl, 0·01 M phosphate, pH 7·1) and any bound antibody traced with fluorescein isothiocyanate-conjugated goat anti-rabbit-globulin. This had a fluorescein to protein molar ratio of 3·6 and predominant reactivity against IgG; before use it was absorbed several times with human tissue homogenates and diluted 1 in 4 so that by itself it gave no staining whatever of the various glandular tissues. It was applied for 30 minutes to the sections, which were then rinsed and washed with buffered saline as before. After mounting in buffered glycerol, the stained sections were examined by darkground ultraviolet fluorescence microscopy with a colourless barrier filter.

Specificity of any immunofluorescent staining was established by its limitation to mucous epithelia, and its absence with normal pre-immune rabbit serum or with the appropriately absorbed immune serum. The presence of any acid mucopolysaccharide-secreting epithelium in the various tissues was established in all cases by staining parallel sections with alcian blue at pH 2·8.

### RESULTS

The findings are summarized in Table I, which shows that the antiserum absorbed with only the red cells and human kidney and lung stained all the mucous epithelia, indicating some common antigenicity; reaction was strongest with colon and ovarian mucinous cystadenoma. After the absorption with bronchus, only the epithelium of the mucinous cystadenomas and of the normal colon could be stained (Fig. 1 and 2). There was no intestinal-specific staining of the serous cystadenomas or of any of the cystadenocarcinomas.

Absorption of the antiserum by the mucinous cystadenoma wall homogenate combined with freeze-dried mucin completely inhibited staining of the cystadenomas and substantially reduced that of colon. All specific reactivity of the serum was abolished by the colon homogenate absorptions. It was important not to misinterpret as specific staining inconstant reactivity with concentrated inspissated mucus found in occasional histological preparations (*e.g.* salivary gland); this was obtained equally with pre-immune and immune sera and could not be completely inhibited by the absorptions.

TABLE I.—*Immunofluorescent Staining of Mucous Glandular Epithelia by Anti-intestine Serum after Absorption by Human Tissues*

| | Antiserum absorbed by: AB red cells, kidney and lung homogenates | Antiserum absorbed by: AB red cells, kidney and lung homogenates + bronchus | Antiserum absorbed by: AB red cells, kidney and lung homogenates + cystadenoma | Antiserum absorbed by: colon homogenate | Preimmune serum absorbed by kidney and lung homogenate |
|---|---|---|---|---|---|
| Colon | +++ | ++ | + | − | − |
| Colon carcinoma, Bronchus, Salivary gland, Pancreas, Bile duct | ++ | − | − | − | − |
| Prostate, Cervix | + | − | − | − | − |
| Ovarian mucinous cystadenoma | +++ | ++ | − | − | − |
| Ovarian serous cystadenoma | + | − | − | − | − |
| Ovarian mucinous cystadenocarcinoma | + | − | − | − | − |
| Ovarian serous cystadenocarcinoma | − | − | − | − | − |

### DISCUSSION

The organ-specificity of this antiserum has been established previously, and when appropriately absorbed, its staining reactivity is limited to mammalian intestinal mucin without cross-reaction with other normal tissues (Nairn *et al.*, 1962*a*; de Boer *et al.*, 1969). In this study we have demonstrated cross-reactivity with the epithelium of three benign mucinous cystadenomas indicating intestinal antigenicity here. The fact that reactivity with colonic mucosa could be only partly suppressed by absorption of the antiserum with cystadenoma suggests that at least two antibodies may be concerned in the colon staining. One antibody apparently cross-reacts with colon and cystadenoma, and the other is against an exclusively intestinal antigen.

The immunofluorescence and absorption studies confirm the well known fact that mucinous cystadenomas contain some mucins which have a general distribution in the mucous epithelia of the body, and also reveal that there is at least one mucin undetected in all the other tissues examined except colon mucosa. It might be speculated that the antigen found only in colon is a reflection of intestinal maturation which has no counterpart in the ovarian tumours. A large series of cystic ovarian tumours should be investigated in this way to decide if this antigenic pattern is a general phenomenon.

The finding of McNeil *et al.* (1969) of colon cancer specificity exhibited by an antiserum to ovarian mucinous cyst fluid has not been investigated in this study though we have reconfirmed the lack of intestinal specificity in adenocarcinomas of the colon (Nairn *et al.*, 1962*b*). The failure to demonstrate intestinal antigenicity in mucinous cystadenomas in the initial studies with this antiserum is perhaps attributable to the relatively primitive immunofluorescence techniques employed 10 years ago. In particular the present procedure of snap-freezing of tissue blocks in liquid nitrogen-isopentane for cryostat sectioning has vastly improved preservation of tissue morphology and antigenicity. The absence of intestinal antigenicity in the mucinous cystadenocarcinomas is presumably yet another example of loss of organ-specificity, which appears to be a general feature of malignancy (Nairn *et al.*, 1966).

We wish to thank Dr. W. G. R. M. de Boer for help with preliminary studies, and Mrs. J. McNaughton and Mr. B. Roudnew for technical assistance. The work was supported by grants from the Anti-Cancer Council of Victoria, the National Health and Medical Research Council and the Medical Research Council of Canada.

### REFERENCES

CARIKER, M. AND DOCKERTY, M.—(1954) *Cancer, N.Y.*, **7**, 302.

DE BOER, W. G. R. M., FORSYTH, A. AND NAIRN, R. C.—(1969) *Br. med. J.*, iii, 93.

EVANS, R. W.—(1966) ' Histological Appearances of Tumours ', 2nd edition. Edinburgh (Livingstone).

MCNEIL, C., LADLE, J. N., HELMICK, W. M., TRENTELMAN, E. AND WENT, M. W.—(1969) *Cancer Res.*, **29**, 1535.

NAIRN, R. C.—(1969) ' Fluorescent Protein Tracing ', 3rd edition. Edinburgh (Livingstone).

NAIRN, R. C., FOTHERGILL, J. E., MCENTEGART, M. G. AND PORTEOUS, I. B.—(1962*a*) *Br. med. J.*, i, 1788.

NAIRN, R. C., FOTHERGILL, J. E., MCENTEGART, M. G. AND RICHMOND, H. G.—(1962*b*) *Br. med. J.*, i, 1791.

NAIRN, R. C., GHOSE, T. AND TANNENBERG, A. E. G.—(1966) *Br. J. Cancer*, **20**, 756.

TACHIBANA, T.—(1929) *Jap. J. Obstet, Gynec.*, **12**, 190. Cited by Cariker and Dockerty (1954).